# Systemic Blockade of Dopamine D2-Like Receptors Increases High-Voltage Spindles in the Globus Pallidus and Motor Cortex of Freely Moving Rats

**DOI:** 10.1371/journal.pone.0064637

**Published:** 2013-06-05

**Authors:** Chen Yang, Shun-Nan Ge, Jia-Rui Zhang, Lei Chen, Zhi-Qiang Yan, Li-Jun Heng, Tian-Zhi Zhao, Wei-Xin Li, Dong Jia, Jun-Ling Zhu, Guo-Dong Gao

**Affiliations:** 1 Department of Neurosurgery, Tangdu Hospital, Fourth Military Medical University, Xi'an, People’s Republic of China; 2 Department of Pathology, Tangdu Hospital, Fourth Military Medical University, Xi'an, People’s Republic of China; 3 Department of Neurosurgery, Urumqi General Hospital of Lanzhou Military Command, Urumqi, People’s Republic of China; Duke University Medical Center, United States of America

## Abstract

High-voltage spindles (HVSs) have been reported to appear spontaneously and widely in the cortical–basal ganglia networks of rats. Our previous study showed that dopamine depletion can significantly increase the power and coherence of HVSs in the globus pallidus (GP) and motor cortex of freely moving rats. However, it is unclear whether dopamine regulates HVS activity by acting on dopamine D_1_-like receptors or D_2_-like receptors. We employed local-field potential and electrocorticogram methods to simultaneously record the oscillatory activities in the GP and primary motor cortex (M1) in freely moving rats following systemic administration of dopamine receptor antagonists or saline. The results showed that the dopamine D_2_-like receptor antagonists, raclopride and haloperidol, significantly increased the number and duration of HVSs, and the relative power associated with HVS activity in the GP and M1 cortex. Coherence values for HVS activity between the GP and M1 cortex area were also significantly increased by dopamine D_2_-like receptor antagonists. On the contrary, the selective dopamine D_1_-like receptor antagonist, SCH23390, had no significant effect on the number, duration, or relative power of HVSs, or HVS-related coherence between M1 and GP. In conclusion, dopamine D_2_-like receptors, but not D_1_-like receptors, were involved in HVS regulation. This supports the important role of dopamine D_2_-like receptors in the regulation of HVSs. An siRNA knock-down experiment on the striatum confirmed our conclusion.

## Introduction

Various neuronal network oscillatory activities are associated with specific brain states [1], [2], [3]. The abnormal function of those activities is thought to underlie a host of human neurological and psychiatric disorders [4]. High-voltage spindles (HVSs), which exhibit a characteristic spike-and-wave pattern and an oscillation frequency ranging between 5 and 13 Hz [5], have been reported to appear spontaneously and widely in the cortical–basal ganglia (BG) networks of rats [3], [4], [6], [7], [8], [9], [10]. Previous studies have shown that interference with striatal dopaminergic transmission can increase the incidence of cortical HVSs, which indicates the possible role of dopamine in the modulation of HVSs [3], [7], [11], [12], [13], [14]. Although the relationship between dopamine and HVSs has been extensively studied using different strains of rats, the function of HVSs is still in debate [4], [6], [15], [16], [17].

In our recent study, we found that dopamine depletion significantly increased the power and coherence of HVSs in the globus pallidus (GP) and motor cortex of freely moving rats [18]. This indicates that dopamine plays a crucial role in the regulation of HVS activity throughout the cortical–BG circuit. Dopamine acts by binding to specific membrane receptors [19] that belong to the G protein-coupled receptors, otherwise known as the seven-transmembrane domain receptors. Five distinct dopamine receptors have been isolated, characterized and subdivided into two subfamilies, D_1_- and D_2_-like, on the basis of their biochemical and pharmacological properties. The D_1_-like subfamily comprises D_1_ and D_5_ receptors, while the D_2_-like subfamily includes D_2_, D_3_ and D_4_ receptors [20]. However, it is unclear whether dopamine regulates HVS activity by acting on dopamine D_1_-like receptors or D_2_-like receptors.

In the present study, we employed local-field potential (LFP) and electrocorticogram (ECoG) methodologies to simultaneously record the oscillatory activities in the GP and primary motor cortex (M1) in freely moving Sprague-Dawley rats. The number, duration and power of HVSs, and HVS-related coherences between M1 and GP, were calculated after systemic administration of dopamine receptor antagonists or saline.

## Materials and Methods

### 1. Animals and Groups

All experiments were approved by the Institutional Animal Care and Use Committee of the Fourth Military Medical University (Permit Number 11018), and carried out in accordance with the national and institutional rules. We minimized the number of animals used and the extent of animal suffering during all experiments. Forty-eight male Sprague-Dawley rats (270–310 g) supplied by the Laboratory Animal Center of the Fourth Military Medical University were used for the study. Twenty-four rats were randomly divided into three age-matched groups for drug treatments (Table 1). Another 24 rats were divided randomly into three groups (eight rats per group) for the siRNA knock-down experiment, with each group receiving either AAV-shSCR (control vector), AAV-shRNA-D_1_ or AAV-shRNA-D_2_ via stereotaxic injection. Animals were housed in standard housing conditions at a constant temperature (22±1°C) and humidity level (relative, 50% –60%), with a 12-hour light/dark cycle (light period 07∶00–19∶00). Water was available ad libitum and food intake was limited to 10–20 g/day to maintain constant animal weight.

**Table 1:** Experimental groups and drug treatments used in the present study.

Group	Drug treatment (IP)
Group 1 (n = 8)	Saline
	SCH23390 1.0 mg/kg
	SCH23390 3.0 mg/kg
Group 2 (n = 8)	Saline
	Raclopride 1.0 mg/kg
	Raclopride 3.0 mg/kg
Group 3 (n = 8)	Saline
	Haloperidol 1.0 mg/kg
	Haloperidol 3.0 mg/kg

The saline or drugs were given IP 30 min before recordings. Each rat was tested with different doses in a counterbalanced order, with an interval of at least 10 days. IP, intraperitoneal injection.

### 2. Drugs

Raclopride and SCH23390 were purchased from Sigma-Aldrich (St. Louis, MO, USA) and freshly prepared in sterilized saline. Haloperidol (Sigma-Aldrich) was dissolved in saline containing 5% acetic acid, and the pH was adjusted to 6.0 with 1 M NaOH. Raclopride, SCH23390 and haloperidol were injected intraperitoneally (IP) at 1.0 or 3.0 mg/kg. Each rat was tested with different doses in a counterbalanced order, with an interval of at least 10 days (Table 1). All the drugs were injected at a volume of 1.0 ml/kg animal weight. Saline (containing 5% acetic acid, pH = 6.0) injections of equal volumes were used for control recordings. The injections were performed at the same times during the 9∶00 to 17∶00 period to minimize circadian variations.

### 3. Surgery

The animals were deeply anesthetized with sodium pentobarbital (35 mg/kg, IP) and ketamine (60 mg/kg, IP). Each rat was fixed in a stereotaxic apparatus (David Kopf Instruments, Tujunga, CA, USA) and the dorsal surface of the skull was then exposed and cleaned. One stainless steel screw (0.8 mm) was implanted into the skull overlying the right frontal regions of the cortex in order to record ECoG from the M1 cortex (AP +2.00 mm, ML −2.25 mm). One additional screw was driven into the bone along the midline, 3 mm anterior to the bregma, to act as the reference electrode for ECoG signals. Semi-micro concentric bipolar electrodes (customized SNEX-100, Rhodes Medical Instruments, Tujunga, CA, USA) were implanted into the right GP (AP: −1.00 mm, ML: −2.80 mm, DV: −6.40 mm). Finally, the two screws and electrodes were fixed to the skull of each rat with dental cement. After surgery, animals were given antibiotics and housed individually in cages for a 2-week recovery period prior to the first recording session.

### 4. siRNA Knock-down Experiment

#### 4.1 Construction of siRNA expression vectors

Expression of hairpin siRNA was based on the pAKD.CMV.bGlobin.eGFP.U6.shRNA vector (Neuron Biotech Co. Shanghai, China) with U6 promoter. Briefly, two 21-nucleotide sequences for the targeting of genes Drd1 (NM_012546, bp 227–245, GGTGACCAACTTCTTTGTC) and Drd2 (NM_012547, bp 451–469, CTACTATGCCATGCTGCTC) were selected in accordance with a previous publication [21]. A control sequence (TTCTCCGAACGTGTCACGT) was chosen on the basis of a previous report [22]. The 55-oligonucleotides for hairpin siRNA, which contained sense and anti-sense targeting sequences were then designed, with a loop (CTCGAG) in the middle and Agel/EcoRI overhangs. The oligonucleotides were synthesized by Invitrogen Life Technologies and were annealed, and ligated into an Agel/EcoRI-linearized pAKD.CMV.bGlobin.eGFP.U6.shRNA vector.

#### 4.2 Production of recombinant virus

The viral package service was provided by Neuron Biotech. The methods for packaging plasmids in recombinant AAV using a triple-transfection, helper-free method was based on a previously reported protocol [23], [24]. Briefly, HEK293 cells were transfected with pAAV-shRNA, pHelper and pAAV-RC plasmids using a standard calcium phosphate method. The vector preps used in the study ranged from 5.1×10^12^ to 1.1×10^13^ vector genomes/ml, and equal dose comparisons were made by normalizing titers with the diluents (lactated Ringer’s solution; Baxter, Deerfield, IL, USA).

#### 4.3 Virus injection

The viruses were administered into the striatum via a stereotaxic injection. Briefly, the rats were anesthetized and placed in a stereotaxic frame. Using a precision Hamilton micro-syringe with a 26 G needle, a total of 4 µl viral solution was bilaterally injected into the ventral and dorsal striatum (AP: −0.90 mm, ML: ±4.50 mm, DV: −5.00 mm and −6.50 mm ventral to the bregma). Viruses were infused at a rate of 0.1 µl/min for 10 min (final volume 1.0 µl/site) and the Hamilton micro-syringe was held in place for an additional 10 min before being slowly withdrawn. Further experiments were performed at least 1 week after surgery.

#### 4.4 Western blotting

The striatum region surrounding the siRNA injection site was dissected, homogenized in buffer and incubated at 4°C with shaking for 20 min. The protein concentration of the supernatants was quantified using the Bradford reaction. Equal amounts of total protein were subjected to SDS-PAGE and transferred to nitrocellulose membranes. Western blotting was performed using standard protocols. Membranes were blocked with 5% BSA in Tris-buffered saline–Tween 20, and incubated with a primary antibody against D_1_R (1∶1000; Abcam) or D_2_R (1∶800; Abcam) overnight at 4°C. Secondary antibodies conjugated to IRDye TM 800 (1∶20 000 dilution; Rockland) were detected using an Odyssey infrared imaging system (LI-COR).

### 5. Behavioral Tests

A bar test was used to assess catalepsy resulting from dopamine receptor antagonists. The bar test involved placing the forepaws of the rat onto a horizontal bar (1 cm diameter) placed 10 cm above the ground 30 min after the drugs injection. Catalepsy was determined by measuring the time taken for one forepaw to be withdrawn from the bar and touch the floor. The latency cutoff time was set at 180 s.

In the siRNA knock-down experiment, locomotion activity was measured using photo-beam sensor equipment. A 40×40 cm clear wall poly-carbonated cage was divided into nine equal squares by four infrared beams (3×3). The horizontal locomotor activity of the rats was measured in terms of the number of crossing of beams 30 min after the injection of 1 ml/kg saline in dim light conditions lasting for 2 hours.

### 6. Electrophysiology

To allow rats to become familiar with the experimental apparatus, rats were twice placed into the recording environment for 1 h prior to the start of the recordings. On the day of recording, a 20-min period was provided to familiarize the rats with the cages. A grounded plate was placed under the recording cages to reduce electromagnetic interference. The recordings were started at a fixed time (during 9∶00 to 17∶00) 30 min after the drug or saline injections in order to minimize the circadian variability of arousal. During the recordings, the rats were allowed to move freely within the cages and behavioral changes were monitored with video tracking. We used the Powerlab 16/30 recording system (ADInstruments, Sydney, Australia) with a band-pass filter of 0.15∼70 Hz and a sampling frequency of 400 Hz per channel. Digitized data were stored on hard disks for offline analysis.

The HVS discrimination was based on a previously reported technique [5]. Activity was considered to be an HVS period when two main features were exhibited: (1) a spike-and-wave pattern; and (2) an oscillation frequency ranging between 5 and 13 Hz. Both criteria were used to detect the beginning and end of HVS episodes. For frequency criteria, a threshold on the instantaneous power spectral density (PSD) of the LFPs was used. The PSD was computed every 100 ms using an overlapping sliding window with a length of 200 sample points (0.5 s). The instantaneous averaged PSD in the 5–13 Hz range was computed for each time step. Its value was deemed to have significantly increased when it passed the threshold (T), which was computed as T = mean +3 SD of the instantaneous PSD values over the whole recording session. Over-the-threshold time steps determined preliminary epochs. A pattern criterion was assessed manually. Preliminary epochs in which the LFPs showed no typical spike-and-wave pattern were rejected. After HVSs were identified, the statistical analyses of number and duration of HVSs were completed using the data from the LFPs in the GP.

The ECoG and LFP signals were analyzed using custom programs written in Matlab (Mathworks Inc.). Synchronization within neuronal ensembles at each electrode position may be quantified by calculating the relative or absolute power in distinct frequency bands [25]. Here, the PSD for each 30 s artifact-free segment was estimated using Welch's method, with a Hanning window of 1024 samples and an overlap of 512 data points for Fast Fourier Transform calculation. The relative PSD (rPSD) was defined as the ratio of PSD to total power in the 1–70 Hz frequency range [18].

Synchronization between distributed neuronal ensembles at each electrode position over different brain regions can be assessed by calculating coherence. Coherence is a traditional measure of the statistical interdependencies between signals across frequencies [26]. Here, coherences were calculated via Welch's averaged method for a pair of 30 s artifact-free segments, with the same window and overlap as for the PSD calculation. This was defined as:
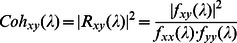
(1)where *f_xy_(λ)* is the cross-spectrum of *x* and *y* at frequency *λ*, *f_xx_(λ)* and *f_yy_(λ)* are the respective auto-spectra of *x* and *y*, and *R_xy_(λ)* is coherency which measures the linear time-invariant relationship between two time-series. Coherence is a bounded measure with a value from 0 to 1, where 0 indicates that there is no linear association and 1 indicates a perfect linear association. Coherence was considered to be significant if it was above the 95% confidence limit (CL). The CL was estimated by:
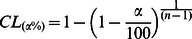
(2)where α = 95 and 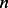 is the number of epochs used in the estimation of auto- and cross-spectra.

### 7. Histology

After all the recordings were completed, rats were deeply anesthetized with sodium pentobarbital (40 mg/kg body weight), a current (50 µA, 10 s) was passed through the tips of the electrodes for LFP recordings in order to create small marker lesions. Rats were then perfused transcardially with 200 ml of 5 mM sodium phosphate-buffered 0.9% (w/v) saline (pH 7.3), followed by further perfusion with 500 ml of 4% (w/v) paraformaldehyde in 0.1 M phosphate buffer. The brains were removed, cut into several blocks and postfixed at 4°C overnight with the same fixative. After cryoprotection with 30% (w/v) sucrose in 0.1 M phosphate buffer, the brain blocks were cut into 30-µm-thick transverse sections on a cryostat. Those sections encompassing the GP were counterstained with cresyl violet for structural identification and electrode placement verification. In addition, GFP expression was examined by fluorescence microscopy in the slices containing the virus injection sites (in the stratum region) in order to assess placement accuracy.

### 8. Statistical Analysis

The number of HVS episodes in the 2-hour recording session following the drug or saline injection was counted. Twelve of the recorded segments (30 s artifact-free segments) that contained HVS episodes were randomly selected for each rat; these segments were distributed over the entire 2-hour recording session. Then, the average values for HVS duration and coherence in the frequency range associated with the HVS activity were obtained by averaging the values from the twelve selected recorded segments. For standard comparisons, the mean and SD of the average values were obtained for each rat. All data are presented in the form of mean ± SD. Statistical analyses were performed using SPSS 17.0. A two-way analysis of variance (ANOVA) was used to assess the overall effects of drugs vs. doses on the number, duration and power of HVSs, and the coherence relating to the HVSs. This was followed by a one-way ANOVA carried out to determine significance. Post-hoc Tukey tests were subsequently performed to determine specific differences between groups. A one-way ANOVA and post-hoc Tukey tests were also conducted on the siRNA knock-down experimental data. A P-value of <0.05 was accepted as statistically significant.

## Results

### 1. General Features of HVSs

**Figure 1:**
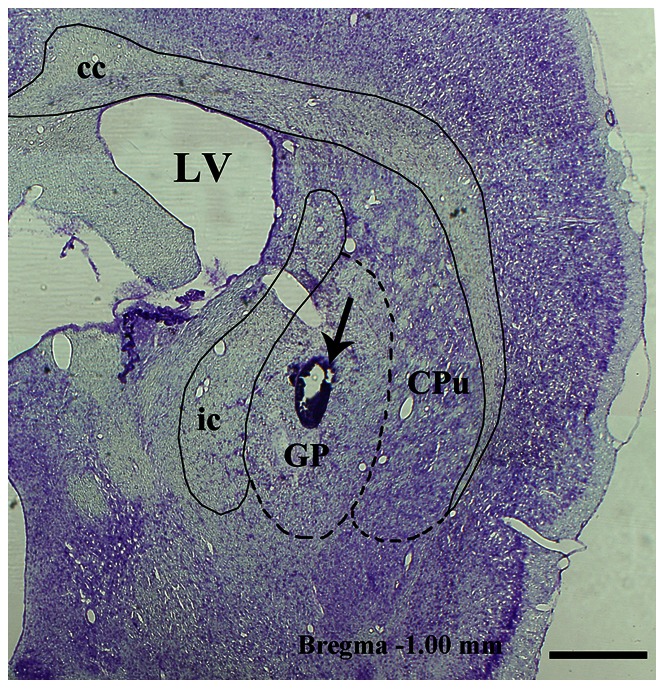
Electrode placement in the GP was verified by observation of the coronal sections counterstained with cresyl violet using light microcopy. The arrow points to the small marker lesions, indicating the location of the electrode tip for LFP recording. Cc, corpus callosum; CPu, caudate putamen; GP, globus pallidus; ic, internal capsule; LV, lateral ventricle. Scale bar = 1 mm.

**Figure 2:**
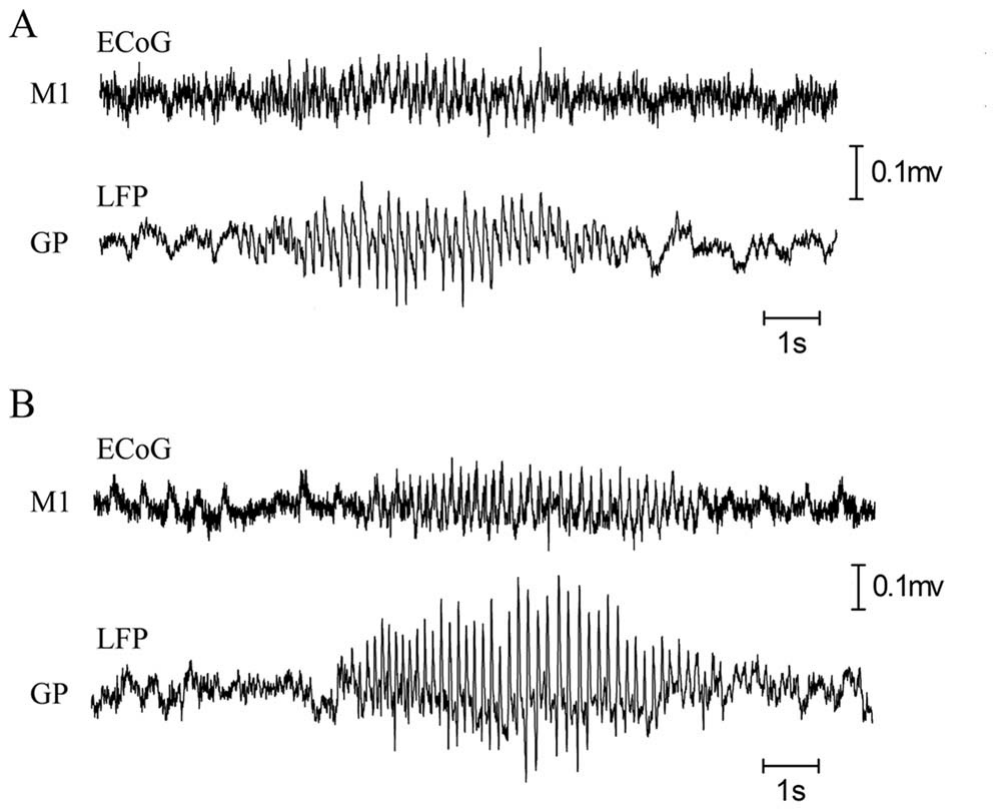
Representative examples of spontaneous high-voltage spindles (HVSs) recorded with ECoG in the M1, and LFP in the GP of saline-treated rats (A) and raclopride-treated rats (B). In saline-treated rats, paroxysmal HVS activities were prominent in the GP area and to a small extent in the M1 cortex. In raclopride-treated rats, paroxysmal HVS activities were prominent in both the M1 cortex and the GP. The HVS episodes were longer and the power was greater following the injection of raclopride. ECoG, electrocorticogram; M1, primary motor cortex; LFP, local-field potential; GP, globus pallidus.

Histological detection confirmed that the LFP-recording electrodes were accurately located in the GP of rats (Fig. 1). The HVS activities for all rats (that had either the saline or drug injections) were recorded simultaneously in the M1 cortex and GP during immobile waking (Fig. 2). During HVS epochs, there was no visible movement except vibrissae twitching. The HVS activities disappeared as soon as the animal moved, as observed by video monitoring.

### 2. Behavioral Changes

**Figure 3:**
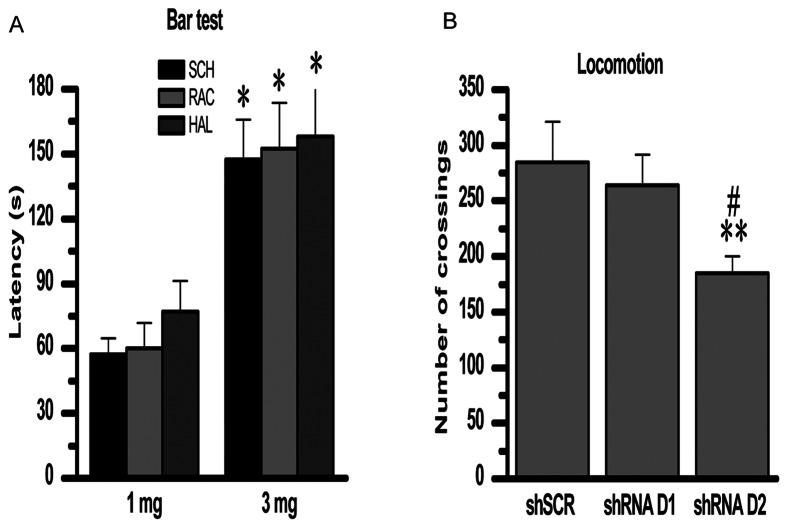
Behavioral changes after the systemic injection of dopamine receptor antagonists or stereotaxic injection of AAV-shRNA into the striatum. A: a bar test was used to assess catalepsy induced by either SCH23390, raclopride or haloperidol. B: locomotion activity was measured using photo-beam sensor equipment. SCH, SCH23390; RAC, raclopride; HAL, haloperidol. *P<0.05 vs. lower dosage. **P<0.01 vs. shSCR. ^#^Significant difference between shRNA D_1_ and shRNA D_2_.

Rats injected with saline readily moved from their awkward position when placed on the bar apparatus. In contrast, all rats injected with drugs (SCH23390, raclopride, and haloperidol) at dosages of 1 mg/kg and 3 mg/kg demonstrated catalepsy. These rats maintained their position on the bar apparatus for at least 57 s. The higher dose (3 mg/kg) was more effective in inducing catalepsy than the lower dose (1 mg/kg) (F [1, 42] = 309.57, n = 8, P<0.001; see Fig. 3A).

Locomotion activity was measured two weeks after the injection of AAV-shRNA. There was no significant difference between the control group and the AAV-shRNA-D_1_ group in terms of locomotion (P = 0.31). In contrast, there was a significant decrease of locomotion in the AAV-shRNA-D_2_ group compared with the control group and the AAV-shRNA-D_1_ group (F [2], [21] = 28.039, n = 8, P<0.001; post-hoc Tukey test, both P<0.001; see Fig. 3B).

### 3. Effect of Selective Dopamine D_1_-like Receptor Antagonist, SCH23390, on HVSs

**Figure 4:**
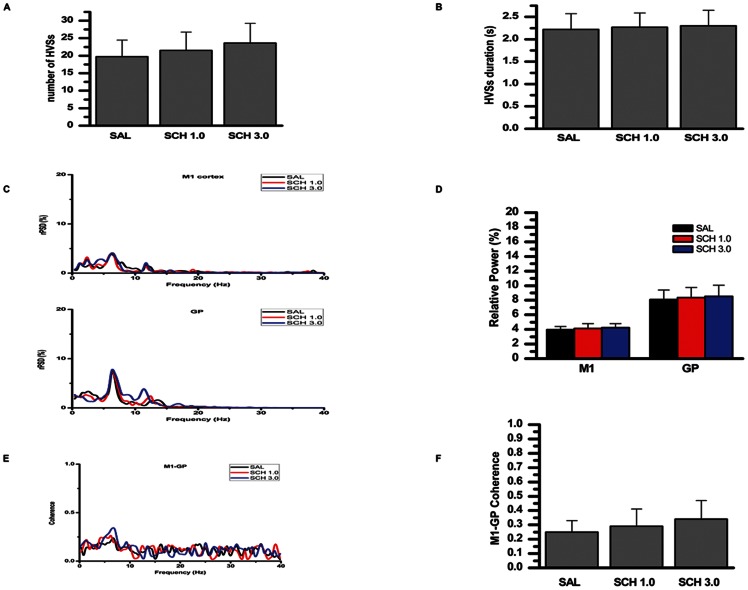
Effects of systemic injection of a selective dopamine D_1_-like receptor antagonist, SCH23390, on the number, duration, and relative power of HVSs, and the HVS-related coherence between the M1 and GP. A: number of HVSs. B: duration of HVSs. C: representative averaged relative power spectral density curve of the M1 cortex (ECoG), and the GP LFP recorded from saline-treated rats (black lines) and SCH23390-treated rats (red or blue lines; the proportion in the 40–70 Hz range was omitted from the curve because of their near-to-zero values). D: relative power of both the M1 cortex and GP LFP in the frequency bands associated with HVS oscillations. E: averaged coherence curves of the M1–GP pair in the saline-treated rats (black lines) and SCH23390-treated rats (red or blue lines; the proportion in 40–70 Hz range was omitted from the curve for their near-to-zero values). F: coherence values relating to the HVSs. SAL, saline; SCH 1.0, SCH23390 1.0 mg/kg; SCH 3.0, SCH23390 3.0 mg/kg; HVSs, high-voltage spindles; M1, primary motor cortex; GP, globus pallidus.

There was no significant effect of SCH23390 on the number of HVSs compared with saline-treated rats (F [2], [21] = 1.111, n = 8, P = 0.348; see Fig. 4A). Nor was there a significant effect on the duration of HVSs compared with saline-treated rats (F [2], [21] = 0.099, n = 8, P = 0.907; see Fig. 4B).

**Figure 5:**
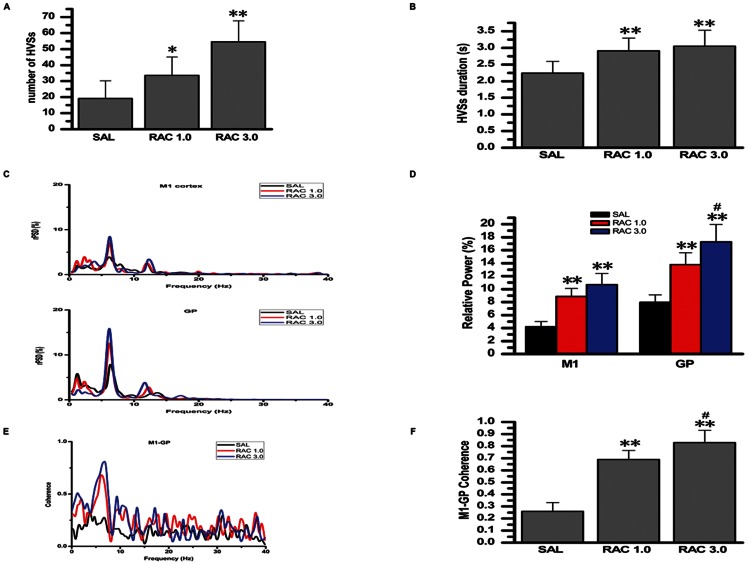
Effects of systemic injection of a selective dopamine D_2_-like receptor antagonist, raclopride, on the number, duration, and relative power of HVSs, and the HVS-related coherence of the M1–GP pair. A: number of HVSs. B: duration of HVSs. C: representative averaged relative power spectral density curve of the M1 cortex (ECoG), and of GP LFP recorded from saline-treated rats (black lines) and raclopride-treated rats (red or blue lines). D: relative power of both the M1 cortex and GP LFP in the frequency bands associated with HVS oscillations. E: averaged coherence curves of the M1–GP pair in the saline-treated rats (black lines) and raclopride-treated rats (red or blue lines). F: coherence values relating to the HVSs. SAL, saline; RAC 1.0, raclopride 1.0 mg/kg; RAC 3.0, raclopride 3.0 mg/kg; HVSs, high-voltage spindles; M1, primary motor cortex; GP, globus pallidus.*P<0.05 and **P<0.01 vs. saline. ^#^Significant difference between RAC 1.0 and RAC 3.0.

**Figure 6:**
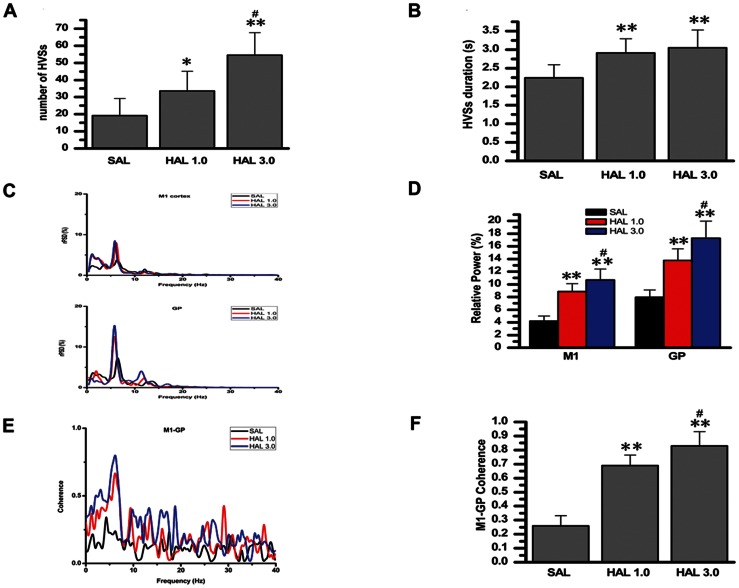
Effects of systemic injection of a non-selective dopamine D_2_-like receptor antagonist, haloperidol, on the number, duration, and relative power of HVSs, and HVS-related coherence of the M1–GP pair. A: number of HVSs. B: duration of HVSs. C: representative averaged relative power spectral density curve of the M1 cortex (ECoG), and of GP LFP recorded from the saline-treated rats (black lines) and raclopride-treated rats (red or blue lines). D: relative power of both the M1 cortex and GP LFP in the frequency bands associated with HVS oscillations. E: averaged coherence curves of the M1–GP pair in the saline-treated rats (black lines) and haloperidol-treated rats (red or blue lines). F: coherence values relating to the HVSs. SAL, saline; HAL 1.0, haloperidol 1.0 mg/kg; HAL 3.0, haloperidol 3.0 mg/kg; HVSs, high-voltage spindles; M1, primary motor cortex; GP, globus pallidus. *P<0.05 and **P<0.01 vs. saline. ^#^Significant difference between HAL 1.0 and HAL 3.0.

**Figure 7:**
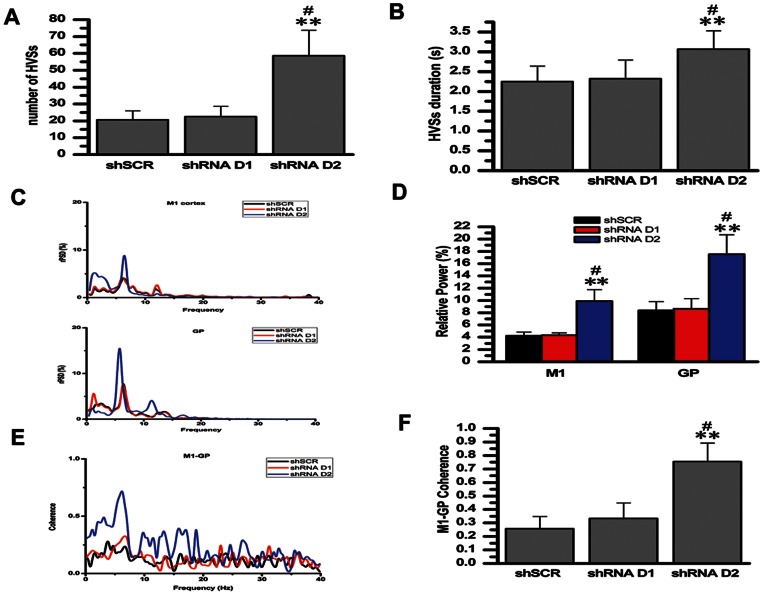
Effects of siRNA knock-down on the number, duration and relative power of HVSs, and the HVS-related coherence of the M1–GP pair. A: number of HVSs. B: duration of HVSs. C: representative averaged relative power spectral density curve of the M1 cortex (ECoG) and of the GP LFP recorded from the rats injected with: AAV-shSCR (black lines), AAV-shRNA-D_1_ (red lines) and AAV-shRNA-D_2_ (blue lines). D: relative power of both the M1 cortex and GP LFP in the frequency bands associated with HVS oscillations. E: averaged coherence curves of the M1–GP pair in the rats injected with: AAV-shSCR (black lines), AAV-shRNA-D_1_ (red lines) and AAV-shRNA-D_2_ (blue lines). F: coherence values relating to the HVSs. HVSs, high-voltage spindles; M1, primary motor cortex; GP, globus pallidus. *P<0.05 and **P<0.01 vs. AAV-shSCR. ^#^Significant difference between AAV-shRNA-D_1_ and AAV-shRNA-D_2_.

Power spectral density was calculated from the 30-s epochs in which the HVS oscillations were present (Fig. 4C, Fig. 5C, Fig. 6C, Fig. 7C). Relative PSD was plotted as a percentage of the total power between 1 and 70 Hz. Prominent peaks around 7 Hz, which was within the frequency range associated with HVS activity, appeared in the rPSD of both the M1 cortex and the LFP in the GP. There was no significant effect of SCH23390 on the relative power in the frequency bands associated with HVS oscillations in either the M1 cortex or the GP, compared with saline-treated rats (F [2], [21] = 0.483, n = 8, P = 0.624, F [2], [21] = 0.193, n = 8, P = 0.826, respectively; see Fig. 4D).

The coherence between the M1 cortex and the GP in frequency bands associated with HVS oscillations was calculated (Fig. 4E, Fig. 5E, Fig. 6E, Fig. 7E). There was no significant effect of SCH23390 on the coherence values relating to the HVSs between the M1 cortex and GP, compared with saline-treated rats (F [2], [21] = 1.012, n = 8, P = 0.381; see Fig. 4F).

### 4. Effect of Selective Dopamine D_2_-like Receptor Antagonist, Raclopride, on HVSs

Raclopride was associated with a significant increase in the number of HVSs (F [2], [21] = 11.299, n = 8, P<0.001). At doses of both 1.0 mg/kg (P = 0.046) and 3.0 mg/kg (P<0.001), raclopride significantly increased the number of HVSs compared with saline-treated rats. However, there was no significant difference between raclopride doses of 1.0 mg/kg and 3.0 mg/kg (P = 0.085; Fig. 5A).

There was a significant effect of raclopride on the duration of HVSs (F [2], [21] = 9.282, n = 8, P = 0.001). Raclopride at doses of 1.0 mg/kg (P = 0.005) and 3.0 mg/kg (P = 0.002) significantly increased the duration of HVSs compared with saline-treated rats. However, there was no significant difference between raclopride 1.0 mg/kg and 3.0 mg/kg (P = 0.949; see Fig. 5B).

Raclopride had a significant effect on the relative power of the peak in the frequency bands associated with HVS oscillations, both in the M1 cortex (F [2], [21] = 33.145, n = 8, P<0.001) and the GP (F [2], [21] = 48.770, n = 8, P<0.001). At doses of 1.0 mg/kg (P<0.001) and 3.0 mg/kg (P<0.001), raclopride significantly increased the relative power of the peak in rPSD compared with saline-treated rats, in both the M1 cortex and the GP. Raclopride 3.0 mg/kg was more effective in increasing the relative power of the peak in GP than 1.0 mg/kg (P = 0.002; see Fig. 5C, 5D **)**. However, there was no significant difference between raclopride 1.0 mg/kg and 3.0 mg/kg in increasing the relative power of the peak in the M1 cortex (P = 0.11; see Fig. 5C, 5D).

There was a significant effect of raclopride on the coherence values relating to the HVSs between the M1 cortex and the GP, compared with saline-treated rats (F [2], [21] = 66.868, n = 8, P<0.001). Raclopride at doses of 1.0 mg/kg (P<0.001) and 3.0 mg/kg (P<0.001), significantly increased the coherence values between the M1 cortex and the GP, compared with saline-treated rats. Raclopride 3.0 mg/kg was more effective in increasing the coherence values than 1.0 mg/kg (P = 0.046; see Fig. 5E, 5F).

### 5. Effect of Non-selective Dopamine Receptor Antagonist, Haloperidol, on HVSs

There was a significant effect of haloperidol on the number of HVSs (F [2], [21] = 17.776, n = 8, P<0.001). Haloperidol at doses of 1.0 mg/kg (P = 0.048) and 3.0 mg/kg (P<0.001) significantly increased the number of HVSs compared with saline-treated rats. Haloperidol 3.0 mg/kg was more effective in increasing the number of HVSs than 1.0 mg/kg (P = 0.006; see Fig. 6A).

There was a significant effect of haloperidol on the duration of HVSs (F [2], [21] = 8.958, n = 8, P = 0.002). Haloperidol at doses of 1.0 mg/kg (P = 0.009) and 3.0 mg/kg (P = 0. 0.002) significantly increased the duration of HVSs compared with saline-treated rats. However, there was no significant difference between haloperidol 1.0 mg/kg and 3.0 mg/kg (P = 0.783; see Fig. 6B).

There was a significant effect of haloperidol on the relative power of the peak in the frequency bands associated with HVS oscillations, both in the M1 cortex (F [2], [21] = 51.735, n = 8, P<0.001) and the GP (F [2], [21] = 45.568, n = 8, P<0.001). Compared with saline-treated rats, haloperidol at doses of 1.0 mg/kg (P<0.001) and 3.0 mg/kg (P<0.001) significantly increased the relative power of the peak for rPSD in both the M1 cortex and the GP. Haloperidol 3.0 mg/kg was more effective in increasing the relative power of the peak than 1.0 mg/kg (M1 cortex: P = 0.028; GP: P = 0.005; see Fig. 6C, 6D).

There was a significant effect of haloperidol on the coherence values relating to the HVSs between the M1 cortex and the GP, compared with saline-treated rats (F [2], [21] = 98.525, n = 8, P<0.001). Haloperidol at doses of 1.0 mg/kg (P<0.001) and 3.0 mg/kg (P<0.001), significantly increased the coherence values between the M1 cortex and the GP compared with saline-treated rats. Haloperidol 3.0 mg/kg was more effective in increasing the coherence values than 1.0 mg/kg (P = 0.008; see Fig. 6E, 6F).

### 6. Effect of siRNA Knock-down on HVSs

**Figure 8:**
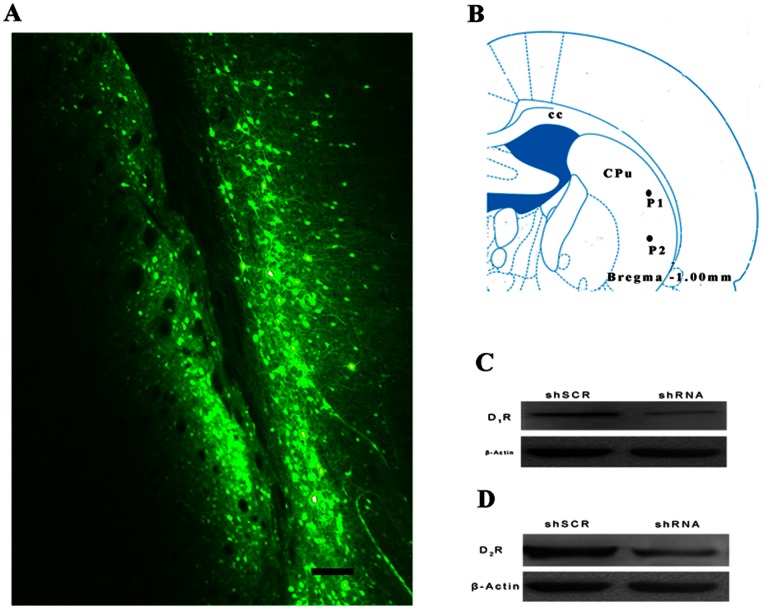
AAV-shRNA mediates dopamine receptors silencing in vivo. A: A coronal section of rat striatum was visualized with fluorescence microscopy 2 weeks after viral injection. GFP expression was confined to the striatum region with some expression occurring in the cortex. B: Two viral injection sites. cc, corpus callosum; CPu, caudate putamen; P1, injection site 1 (ML: +4.5 mm, DV: −5.00 mm); P2, injection site 2 (ML: +4.5 mm, DV: −6.50 mm). Scale bar = 0.1 mm. This drawing was adapted from the atlas by Paxinos and Watson (2005). C: Dopamine D_1_ receptor protein levels in the striatum were detected by Western blotting with β-actin as the internal control. Injection of AAV-shRNA-D_1_R effectively decreased the expression of dopamine D_1_ receptor protein. D: Dopamine D_2_ receptor protein levels in the striatum were detected by Western blotting using β-actin as the internal control. Injection of AAV-shRNA-D_2_R effectively decreased dopamine D_2_ receptor protein expression.

Two weeks after virus injection, GFP expression was examined by fluorescence microscopy in the slices containing the virus injection sites (in the stratum region) in order to assess placement accuracy (see Fig. 8A, 8B).

Western blotting was performed to estimate the down-regulation ability of AAV-shRNA on dopamine receptors. The expression of both dopamine D_1_ and D_2_ receptor proteins was effectively decreased in the AAV-shRNA group compared with the AAV-shSCR group (see Fig. 8C, 8D).

There was a significant difference between injections of AAV-shSCR, AAV-shRNA-D_1_ and AAV-shRNA-D_2_ on the number (F [2], [21] = 37.49, n = 6, P<0.001; see Fig. 7A) and the duration (F [2], [21] = 8.21, n = 8, P = 0.002; see Fig. 7B) of HVSs. There was no significant effect of AAV-shRNA-D_1_ on the number (P = 0.924) and duration (P = 0.94) of HVSs compared with AAV-shSCR. In contrast, the injection of AAV-shRNA-D_2_ significantly increased the number (P<0.001) and the duration (P = 0.004) of HVSs in rats compared with the AAV-shSCR injection. There was significant difference between AAV-shRNA-D_2_ and AAV-shRNA-D_1_ on the number (P<0.001) and the duration (P = 0.009) of HVSs.

There was significant difference between the injections of AAV-shSCR, AAV-shRNA-D_1_ and AAV-shRNA-D_2_ on the relative power in the frequency bands associated with HVS oscillations both in the M1 cortex (F [2], [21] = 61.89, n = 8, P<0.001; see Fig. 7C, 7D) and the GP (F [2], [21] = 44.43, n = 8, P<0.001; see Fig. 7C, 7D). There was no significant effect of AAV-shRNA-D_1_ on the relative power of the peak for rPSD in either the M1 cortex (P = 0.985) or the GP (P = 0.968) compared with AAV-shSCR. In contrast, the injection of AAV-shRNA-D_2_ significantly increased the relative power of the peak in both the M1 cortex (P<0.001) and the GP (P<0.001) compared with the injection of AAV-shSCR. There was a significant difference between AAV-shRNA-D_2_ and AAV-shRNA-D_1_ on the relative power of the peak in both the M1 cortex (P<0.001) and the GP (P<0.001).

There was a significant difference between AAV-shSCR, AAV-shRNA-D_1_ and AAV-shRNA-D_2_ on the coherence values relating to the HVSs between the M1 cortex and the GP (F [2], [21] = 50.586, n = 8, P<0.001; see Fig. 7E, 7F). The AAV-shRNA-D_1_ injection had no significant effect on the coherence values between the M1 cortex and the GP compared with the AAV-shSCR injection (P = 0.34). In contrast, AAV-shRNA-D_2_ significantly increased the coherence values between the M1 cortex and the GP compared with AAV-shSCR (P<0.001). There was a significant difference between AAV-shRNA-D_2_ and AAV-shRNA-D_1_ on the coherence values (P<0.001).

## Discussion

We simultaneously examined the influence of dopamine receptor antagonists on HVSs in the GP and the motor cortex, and analyzed the alteration of the local and inter-regional neuronal synchrony during HVS activity. The main findings of the present study were that dopamine D_2_-like receptor antagonists, but not D_1_-like receptor antagonists, increased the number, duration, and power of HVSs in the GP and M1 cortex, and also increased the HVS-related coherence between the GP and M1 cortex in freely moving rats. A siRNA knock-down experiment was also conducted, which confirmed our conclusion.

Previous research has shown that the systemic administration of haloperidol at the dosage of 2.0 mg/kg effectively increased the incidence and duration of cortical HVSs in young rats, whereas SCH23390 (0–1.0 mg/kg) had no significant effect on cortical HVSs [7], which is consistent with our results. However, the effect of the dopamine antagonist on the synchronization of HVS activity within and between the GP and motor cortex was unclear. Our previous study showed that dopamine depletion can significantly increase the power and coherence of HVSs in the GP and motor cortex of freely moving rats [18]. Our present study confirmed the role of D_2_-like receptors in the regulation of HVSs.

The dopamine pathways in the brain include the: (1) nigrostriatal pathway; (2) mesolimbic pathway; (3) mesocortical pathway; and (4) tuberoinfundibular pathway [20]. Many previous studies have observed that the incidence of cortical HVSs significantly increased after the disturbance of striatal dopaminergic transmission [7], [12], [14], and after dopamine depletion by 6-hydroxydopamine (6-OHDA) injection in the medial forebrain bundle [11], [18]. Using siRNA technology, the present study also showed that HVS activity was enhanced by silencing the dopamine D_2_ receptors but not the D_1_ receptors in the striatum. These findings indicate that the nigrostriatal pathway may play an essential role in modulating HVSs.

It is well known that dopamine D_1_-like and D_2_-like receptors are highly expressed in the BG, but differentially expressed in the direct and indirect pathways [27]. The D_1_-like receptors dominate the “direct” pathway striatal neurons that project to the internal GP, whereas the D_2_-like receptors are found on the “indirect” pathway, which pass through the external GP and project to the subthalamic nucleus [28]. Our previous study showed that the power of HVS activity in the LFP of the GP was increased after dopamine depletion, indicating that local neuronal synchronization in the GP was enhanced during HVS oscillation [18]. The present study observed that dopamine D_2_-like receptor antagonists, but not D_1_-like receptor antagonists, increased the power of HVS activity in the M1 cortex and LFP of the GP, indicating that D_2_-like receptors play a role in the modulation of HVSs. This is in line with previous findings which suggest that dopamine D_2_-like receptors may have a special role in the regulation of BG rhythmic activity via dopamine [29]. As the GP comprises extensive local axon collaterals, and projects to all other BG nuclei, it is considered to be central in propagating and synchronizing oscillatory activity in the cortical–BG networks [30], [31], [32]. This suggests that the systemic injection of D_2_-like receptor antagonists may act via the “indirect” pathway to increase HVSs.

However, dopamine receptors are widely distributed in different forebrain structures such as the cortex, stratum and thalamus [33], [34], [35], [36]. In the past, the existence of significant dopamine innervations in the thalamus had been largely ignored, probably because dopamine innervation of the rodent thalamus was reportedly scant [33], [37]. In fact, the thalamus is made up of multiple nuclei, which relay information from subcortical centers or from other cortical areas, and receive axons containing neuromodulators such as acetylcholine, histamine, serotonin, and the catecholamines adrenaline, noradrenaline and dopamine [33], [37]. Many previous experiments have provided strong evidence to indicate that thalamocortical reverberating loops are a putative motor for HVS generation, and that thalamocortical network activity may generate the HVSs [4], [38], [39], [40], [41]. Consequently, in addition to the nigrostriatal pathway, the thalamocortical network may play a vital role in HVS generation and regulation. However, further exploration is required.

### Conclusions

Our present results suggest that dopamine D_2_-like receptors, but not D_1_-like receptors, are involved in HVS regulation. Our findings support the important role of dopamine D_2_-like receptors in the regulation of HVSs.
